# RNA Sequencing Reveals Alterations and Similarities in Cell Metabolism, Hypoxia and Immune Evasion in Primary Cell Cultures of Clear Cell Renal Cell Carcinoma

**DOI:** 10.3389/fonc.2022.883195

**Published:** 2022-05-11

**Authors:** Adrian Georg Simon, Laura Kristin Esser, Jörg Ellinger, Manuel Ritter, Glen Kristiansen, Michael H. Muders, Thomas Mayr, Marieta Ioana Toma

**Affiliations:** ^1^ Department of Pathology, University Hospital Bonn, Bonn, Germany; ^2^ Department of Pathology, University Hospital Cologne, Cologne, Germany; ^3^ Department of Urology, University Hospital Bonn, Bonn, Germany

**Keywords:** *in vitro* model, cell culture conditions, ccRCC, RNA-Seq, renal cancer, patient-derived model

## Abstract

The treatment of advanced renal cell carcinoma remains a challenge. To develop novel therapeutic approaches, primary cell cultures as an *in vitro* model are considered more representative than commercial cell lines. In this study, we analyzed the gene expression of previously established primary cell cultures of clear cell renal cell carcinoma by bulk (3’m)RNA sequencing and compared it to the tissue of origin. The objectives were the identification of dysregulated pathways under cell culture conditions. Furthermore, we assessed the suitability of primary cell cultures for studying crucial biological pathways, including hypoxia, growth receptor signaling and immune evasion. RNA sequencing of primary cell cultures of renal cell carcinoma and a following Enrichr database analysis revealed multiple dysregulated pathways under cell culture conditions. 444 genes were significantly upregulated and 888 genes downregulated compared to the tissue of origin. The upregulated genes are crucial in DNA repair, cell cycle, hypoxia and metabolic shift towards aerobic glycolysis. A downregulation was observed for genes involved in pathways of immune cell differentiation and cell adhesion. We furthermore observed that 7275 genes have a similar mRNA expression in cell cultures and in tumor tissue, including genes involved in the immune checkpoint signaling or in pathways responsible for tyrosine kinase receptor resistance. Our findings confirm that primary cell cultures are a representative tool for specified experimental approaches. The results presented in this study give further valuable insights into the complex adaptation of patient-derived cells to a new microenvironment, hypoxia and other cell culture conditions, which are often neglected in daily research, and allow new translational and therapeutic approaches.

## Highlights

- Extensive characterization of primary cell cultures compared to the tissue of origin
- Several pathways are alternated in cell culture conditions
- Cell cultures showed signs of pseudohypoxia
- Pathways and genes of therapeutic relevance remained stable
- Primary cells expressed same levels of *VEGFA, VEGFC* and *EGFR in vitro*
- The PD-1 pathway was similarly expressed under cell culture conditions
- Primary cell cultures are feasible *in vitro* tool for specific experimental approaches

## Introduction

Clear cell renal cell carcinoma (ccRCC) accounts for 70-75% of renal cancer entities (1). Since ccRCC is resistant to conventional chemotherapy and radiation, in case of advanced or metastatic disease immune checkpoint inhibitors and tyrosine kinase inhibitors are the current systemic therapeutic approach (2, 3). But only 42% of treated patients respond to these therapeutics, with a median progression-free survival of 11.6 months (4, 5). The exact molecular changes causing ccRCC progression as well as initial or acquired therapy resistance are still unknown.

For decades, cell cultures have been used for the investigation of tumor biology *in vitro* and testing feasibility, effectiveness and toxicity of new drugs. However, the commercially available cell lines mostly used, e.g. 786-O or A498, do not represent models close to the tissue of origin, raising questions about their genetic alterations and changes in metabolism acquired over time, or even their origin to begin with (6–8).

Primary cell cultures as an individual, patient-derived *in vitro* model promise higher similarity to the tissue of origin (9–11). Most current studies confirmed that established primary cells lines of RCC entities match the phenotype of ccRCC or non-neoplastic renal tissue by immunohistochemistry, immunofluorescence (11) or assessment of specific characteristic gene mutations, e.g. the *VHL* gene mutation in ccRCC (12, 13). Proteomic analysis of five paired primary RCC and derived primary cell lines revealed 65% matched spots on mass-spectrometry analysis (14). A further study showed that 27 of 28 investigated proteins have a similar expression in primary cell lines from RCC and matched tissue of origin (15). However, the knowledge about alternated gene expression on a broad spectrum in patient-derived cultures remains sparse. The adaptation mechanisms of cell cultures to a new microenvironment and *in vitro* conditions like hypoxia or a missing extracellular matrix have been rarely investigated, although they potentially affect nearly every experimental research and its results. At the same time, it is widely unknown, which pathways are unaffected by these adaptational alterations and to which extend the cell cultures can therefore be seen as a representative *in vitro* model. To our knowledge, this is the first study investigating at a large scale the gene expression changes of ccRCC primary cell cultures and matched tumor tissue by 3’ mRNA sequencing, exploring specific pathways that are dysregulated or differently expressed under culture conditions as well as identifying pathways which are equally or similarly expressed *in vitro.*

## Material and Methods

All materials, if not stated otherwise, were acquired by Thermo Fisher Scientific (Waltham, USA).

This study was performed with the informed consent of all patients as well as the ethics committee at Bonn University Hospital (No. 219/17).

### Establishing the Primary Cell Line Cultures

Fresh tumor tissue from eight patients was obtained and cultivated as reported before (16). All patients underwent partial or complete nephrectomy for ccRCC at Bonn University Hospital (**Table 1**). In short, approximately 2 cm^3^ tumor tissue as well as non-neoplastic tissue were obtained. The tissue was minced and digested in 10 mL RPMI 1640 containing 200 U/mL collagenase type II, 100 U/mL hyaluronidase type V (Sigma Aldrich, USA) and 2% penicillin/streptomycin at 37°C under constant shaking (150 rpm). After 2 hours, the cells were filtered using sterile 70 µm- and 40 µm-sieves/filter (VWR International, Germany). After the cells were washed twice with DPBS and centrifuged (1000 rpm, 5 min), the supernatant was carefully discarded. The primary cells were cultivated in serum-reduced medium (SRM) (DMEM/F12) containing supplements (5% FBS, 1% penicillin/streptomycin, 10 ng/mL hrEGF (R&D Systems, USA), 10 ng/mL FGF-basic (PeproTech, Germany), 1x B27-supplement, 1x Lipid Mixture 1 (Sigma Aldrich), 1 mM N-Acetyl-Cystein, 4 mM L-Glutamine, 1x non-essential amino acids and 10 mM HEPES (GE Healthcare, UK). All primary cell cultures were tested for mycoplasma contamination on a regular basis.

**Table 1:** Clinicopathological parameters of the included patient specimens.

		N	[%]
Sex	male	4	50%
	female	4	50%
Age (years)	range	50-87	
	median	68	
pT stage	pT1	4	50%
	pT2	0	0%
	pT3	4	50%
ISUP grade	1	1	12.5%
	2	4	50%
	3	3	37.5%

### Gene Expression Analysis by Bulk 3’RNA Seq

RNA from seven ccRCC tissues and established primary cell cultures was extracted using the Universal RNA purification kit (Roboklon, Germany) according to the manufacturer’s instructions. Extracted RNA was further processed by the Next Generation Sequencing Core Facility of the University Hospital Bonn, Germany and analyzed using a HiSeq 2500 v4 sequencer (Illumina). Raw single-read sequencing results were mapped to the human genome (GRCh38) using the alignment program hisat2-2.1.0 (17).

The mapped reads were then processed using samtools (18) and quantified with featureCounts (19). Next, the statistical significance was calculated using the Bioconductor software package DESeq (20). Wald test was applied for p-values of the differentially expressed genes. To correct for multiple testing, the false discovery rate (FDR) was calculated with the Bonferroni method and an FDR cutoff of 0.05 was accepted as significant. Log2 fold shrinkage was performed by applying the apeglm method (21). The principal component analysis (PCA), volcano plot and heatmap were generated using ggplot2 and Enhanced Volcano (22, 23). Subsequent analysis of the differently expressed genes was performed using the Hallmark gene set collection (Molecular Signature Database, Broad Institute). For that, a pre-ranked list with differently expressed genes was called into the Gene Set Enrichment Analysis (GSEA) software with default settings for enriched hallmark gene sets (24). In addition, a pre-selected list -FDR <0.05 and log2FC>2 or log2FC<-2 was called into the database Enrichr (25) to dissect different genetic and functional cellular pathways. The dataset used for this analysis can be found in the online repository Sequence Read Archive under the following accession number: PRJNA803031.

### Quantification of mRNA Expression of Selected Genes

RNA was extracted from primary cell cultures and corresponding tumor tissue using the Universal RNA purification kit (Roboklon, Germany) according to the manufacturer’s instructions. 500 ng RNA were transcribed to cDNA using SuperScript™ II Reverse Transcriptase. Quantitative Real-Time PCR was performed on Viia 7 Real-Time PCR System using iTaq™ Universal SYBR Green Supermix (Bio-Rad Laboratories, USA). Peptidylprolyl isomerase A (PPIA) was used as reference gene. The mRNA expression levels were quantified for: *VHL*, *HIF1A, CA9*, *VEGFA*, *VEGFC* and *CD274*/*PD-L1*. The primers for all selected genes were designed using the primer design tool of National Center of Biotechnology Information (26) and tested for efficiency using a 1:2 dilution series of cDNA of benign renal tissue as well as ccRCC samples obtained from the Department of Urology at Bonn University Hospital. Levels of mRNA expression were quantified and compared between the tissue of origin and the derived primary cell culture using ΔΔC_T_ method (27). Conditions and qPCR primer sequences are provided in **Supplementary Table 1**.

### Immunohistochemical (IHC) Staining of Tissues of Origin and Derived Primary Cell Cultures

For immunohistochemical staining, primary cells were detached with trypsin-EDTA, washed and centrifuged. The cell pellet was resuspended in 300 µL Richard-Allan Scientific HistoGel according to the manufacturer’s instructions, fixed in 4% PFA and embedded in paraffin according to routine protocols at the Institute of Pathology, University Hospital Bonn. IHC stainings were performed on a Medac 480 S Autostainer (Medac, Germany) and on a Ventana BenchMark Ultra Autostainer (Roche Diagnostics, Switzerland). Immunohistochemical staining was assessed using Olympus BX50 microscope (Olympus, Japan). Detailed antibody information is provided in **Supplementary Table 2**.

### Sequencing of *Von Hippel-Lindau* Gene in Primary Cell Cultures and Tumor Tissue of Origin

As reported before, we performed Sanger sequencing for mutations of the three exons of the *Von Hippel-Lindau* gene (*VHL*) in primary cell cultures of eight ccRCC as well as the tumor tissue they derived from (16). Shortly summarized, DNA was extracted using the QIAmp DNA Minikit (Qiagen) according to the manufacturer’s instructions. After amplification of the *VHL* exons (1, 2a, 2b, 3) with a GeneAmp PCR System 9700 (primer sequences: supplementary data), fragment sizes were checked with an agarose gel electrophoresis and the amplicons were purified using the NucleoSpin Gel and PCR Clean-up kit (Macherey-Nagel, Germany). Sanger sequencing was performed by GATC sequencing service (Eurofins Genomics, Germany). To identify *VHL* mutations, ApE Plasmid Editor (Jorgensen Laboratory, USA) and FinchTV (Geospiza Inc., USA) were used. The mutations were checked using The Catalogue of Somatic Mutations in Cancer (COSMIC) (28).

## Results

### 3’mRNA Seq Analysis of ccRCC Primary Cell Cultures and the Tissue of Origin

**Figure 1:**
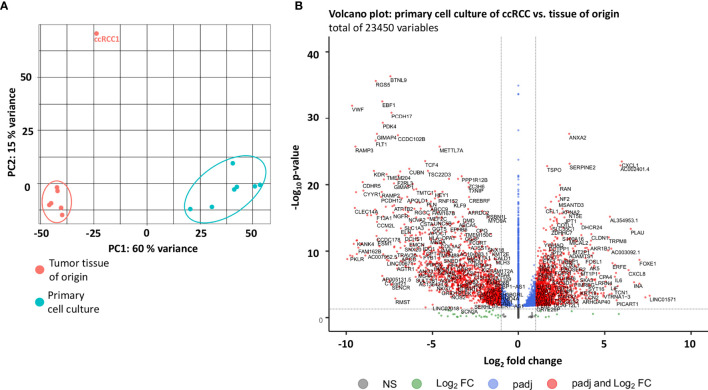
RNA-sequencing: alternated pathways in ccRCC primary cell cultures compared to the tissue of origin. **(A)** PCA plot of mRNA expression profiles in seven ccRCC primary cell cultures compared to the tumor tissue (tissue of origin). Primary cell cultures as well as the tissue of origin clustered, revealing a close genetic similarity within their group, with the exception of ccRCC1 (outlier). This renal cell carcinoma displayed no histological abnormalities, but harbored a *VHL* gene mutation (c.491 A>T, missense substitution, **Supplementary Table 5**). It also displayed slight differences in the top 50 dysregulated genes which a highly aberrant expression of *ALDOB* and *FBG* (see **Figure 2**). **(B)** Volcano plot of the dysregulated genes in primary cell cultures compared to the tissue of origin. A total of 23450 genes displayed genetic alterations. The -Log10 p-value on the y axis displays the statistical significance, Log2 fold change on the x axis reveals over- or reduced expression of the gene. The blue dots represent genes with significant gene alterations within a Log2 fold change of -1 and 1, respectively. The green dots represent gene alterations with p > 0.05 (dashed line). NS = gene alterations, neither significant (p > 0.05) nor with Log2 fold change < -1 or > 1.

RNA sequencing analysis was performed for seven primary cell cultures and seven tumor tissue specimens of ccRCC. A close genetic relationship between primary cell cultures and the tumor tissue of origin was noticed (**Figure 1A**). A total of 24.350 genes in total were dysregulated in primary cell cultures compared to the tissue of origin (**Figure 1B**).

Within these genes, 444 genes were significantly (adjusted p-value < 0.05, Benjamini-Hochberg correction) upregulated (Log2 fold change > 2), 888 genes were downregulated (Log2 fold change < 2). 7.275 genes were expressed with a Log2 fold change between -2 and 2 and were interpreted as equal or similar expression in primary cell cultures and tumor tissue (adjusted p-value < 0.05, Benjamini-Hochberg correction). A list of all genes is available in the **Supplementary Material**.

**Figure 2:**
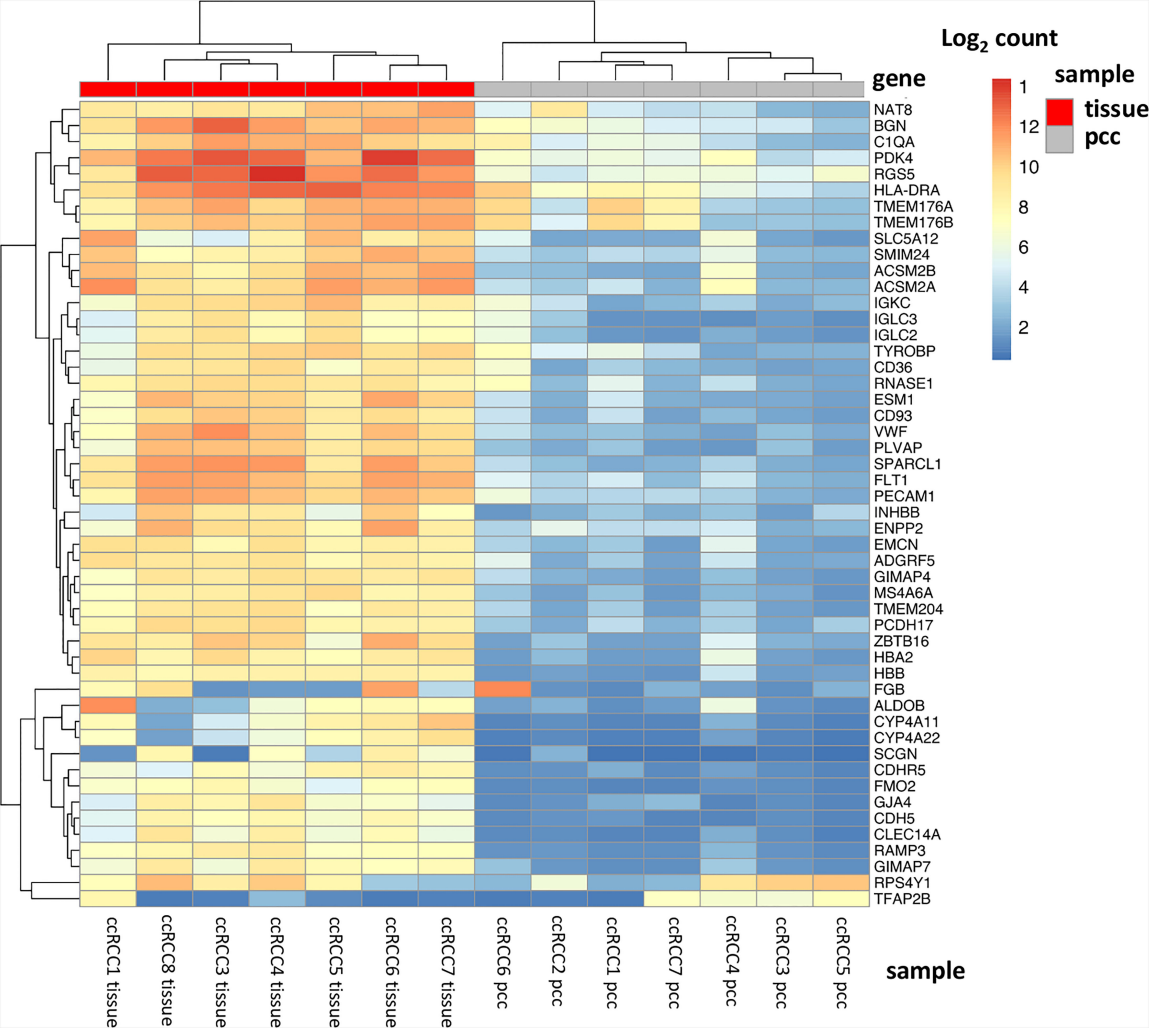
Top 50 dysregulated genes in ccRCC tissue and primary cell cultures. The heatmap displays the top 50 significantly dysregulated genes in seven ccRCC tissue specimens as well as in 7 ccRCC primary cell cultures. Among these genes, there are genes involved in hypoxia signaling and metabolic regulation (*PDK4, RGS5, ALDOB, SLC5A12, ACSM2A, ACSM2B*), in cell adhesion and extracellular matrix (*CDH5, PECAM1, SPARCL1*), angiogenesis (*ESM1*) and immune signaling (*TYROBP*).

The top 50 significantly alternated genes included genes involved in the regulation of cell metabolism (*ALDOB, PDK4, ACSM2A, ACSM2B*) and *SLC5A12*, coding for a lactate-reabsorbing transport protein in the proximal tubule (**Figure 2**). Other differently expressed genes code for cell adhesion molecules as well as interaction with the extracellular matrix (*EMCN, CDH5*, *PECAM1, SPARCL1, PCDH17*, *CDHR5*). *NAT8* is specifically expressed in renal tissue and was dysregulated in ccRCC tissue. Two genes involved in angiogenesis (*ESM1*) and immune signaling (*TYROBP*) were also among the 50 most dysregulated genes in ccRCC cell cultures and tissue.

### Gene Set Enrichment Analysis (GSEA) and Enrichr Analysis of ccRCC Primary Cell Cultures Versus Tumor Tissue

**Figure 3:**
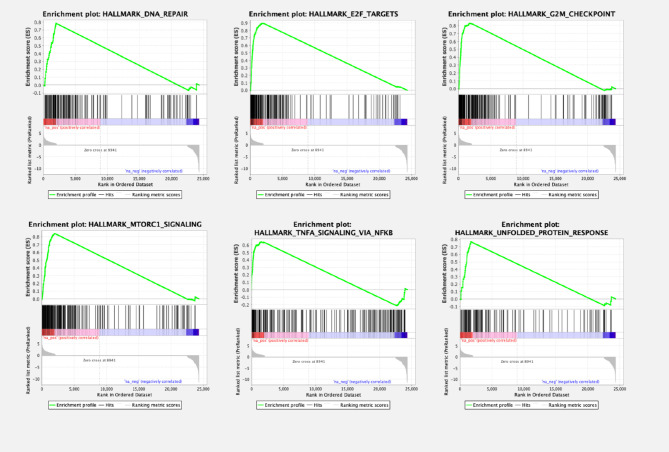
Gene set enrichment analysis (GSEA) plots of enriched pathways in ccRCC primary cell cultures compared to the tissue of origin. Gene set enrichment analysis revealed several pathways being significantly dysregulated, among them pathways involving genes of DNA repair mechanisms. Signaling of E2F and G2M checkpoint regulation, therefore involved in cell cycle regulation, was also dysregulated, as well as MTORC1- and TNFA pathways, other signaling cascades regulating cell proliferation.

To compare biological pathways in ccRCC primary cell cultures and the tissue of origin, a gene set enrichment analysis (GSEA) was performed. Compared to the tissue of origin, the primary cell cultures displayed a total of 28 of 50 gene sets being upregulated. 10 gene sets were significantly enriched at FDR < 25%, 8 gene sets at nominal p-value < 1% and 9 gene sets significantly enriched at nominal p-value < 5% (**Table 2** and **Figure 3**). In cell cultures DNA repair processes, E2F targets, G2/M checkpoints and mitotic spindle formation were enriched, indicating an increased cell cycle progression. Additionally, MTORC1 signaling, TNF-alpha signaling and MYC-targets were overexpressed compared to the tumor tissue *in vivo*.

**Table 2:** Significantly upregulated gene pathways in ccRCC primary cell cultures in gene set enrichment analysis (GSEA).

Pathway	Genes alternated n	p-value
DNA repair	39	p=0.0048
E2F targets	95	p < 0.001
G2/M checkpoint	86	p < 0.001
Mitotic spindle	51	p < 0.001
Glycolysis	47	p < 0.001
MTORC1 signaling	76	p < 0.001
MYC-targets	103	p < 0.001
TNF-alpha signaling	42	p=0.01
unfolded protein response	29	p=0.004

Gene set enrichment analysis (GSEA) revealed 9 gene sets significantly upregulated in ccRCC primary cell cultures compared to the tissue of origin.

**Figure 4:**
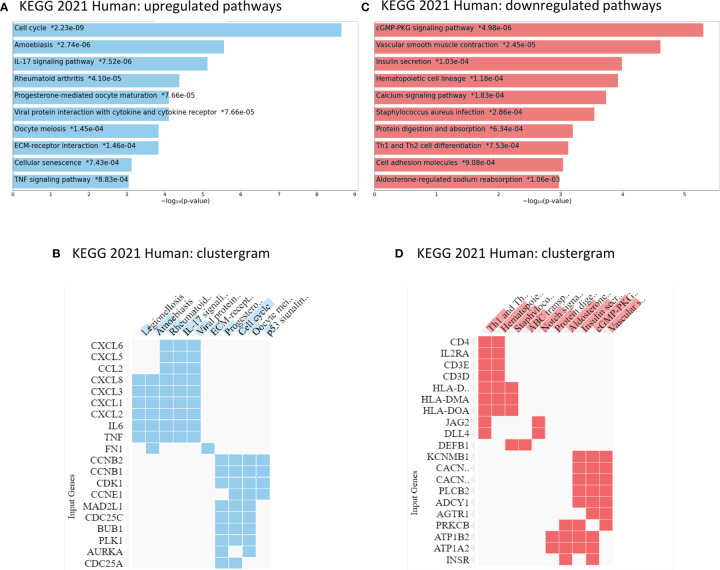
Enrichr analysis of KEGG 2021 Human pathways in ccRCC primary cell cultures: up- and downregulated genes compared to tumor tissue. **(A, B)** Compared to the tissue of origin, ccRCC primary cell cultures displayed an upregulation in cell cycle regulation, IL-17 pathway regulation, other immune response pathways and genes as well as TNF signaling pathways. **(C, D)** The primary cell cultures expressed lower mRNA levels of genes involved in calcium signaling, cell adhesion molecules as well as cGMP-PKG signaling pathways. * p- value, adjusted.

An Enrichr analysis using the KEGG 2021 Human database for pathway assignment of the 444 upregulated genes (log fold change > 2, p adjusted < 0.0.5) confirmed the upregulation of the cell cycle progression as well as TNF signaling (**Figure 4A, B**). Additionally, pathways regulating cellular senescence, IL17-signaling as well as ECM-receptor interaction were increased *in vitro*. Interestingly, the upregulated genes, according to the KEGG 2021 Human assignment, also hold functions in several immune-mediated diseases like rheumatoid arthritis (*CXCL6*, *IL6*, *CXCL8, IL23A, CCL2, CXCL1, CXCL3, CXCL2, TNF, CXCL5*) as well as parasitic infections like amoebiasis (*IL6, CXCL8, CXCL1, PRDX1, FN1, TNF* and other*)*. A list of the Enrichr assignments of the genes is available in the **Supplementary Table 4**. The GSEA enrichment analysis revealed 22 of 50 gene sets being downregulated in primary cell cultures, with no gene set being significantly enriched at FDR < 25% and the nominal p-value <1%, respectively. However, the Enrichr analysis (KEGG 2021 Human database) displayed an assignment of the 888 downregulated genes to pathways involved mainly in cGMP-PKG signaling, calcium signaling, haematopoetic cell lineage pathways, protein digestion and cell adhesion (**Figures 4C, D**; gene assignment **Supplementary Table 4**).

### Enrichr Analysis of Pathways Expressed Equally or Similarly in Primary Cell Cultures and the Tissue of Origin

**Figure 5:**
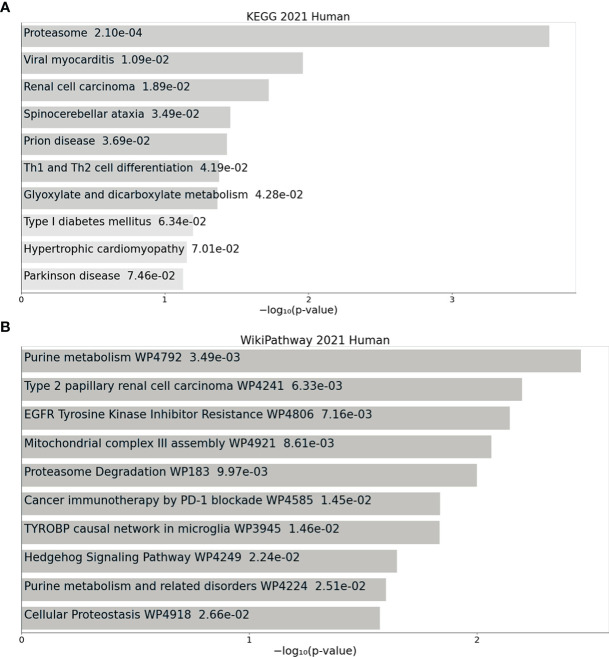
Pathways equally expressed in ccRCC primary cell cultures and the tissue of origin. **(A)** Enrichr analysis revealed that primary cell cultures shared the same mRNA expression levels of pathways involved in proteasome, renal cell carcinoma, several other diseases and glyoxylate and dicarboxylate metabolism (KEGG 2021 Human pathways). **(B)** The primary cell cultures furthermore displayed a very similar expression of genes regulating purine metabolism, resistance mechanisms against EGFR tyrosine kinsase inhibtors and cancer immunotherapy.

7.275 genes were identified which were expressed on equal or similar levels in cell culture conditions to the tissue of origin (see above, p adjusted < 0.05). An Enrichr analysis using KEGG 2021 Human and WikiPathway 2021 Human databases assigned these genes to several central pathways in renal cell carcinoma, proteasome regulation, glyoxylate and decarboxylate metabolism (KEGG 2021 Human) (**Figure 5A**). An alignment with WikiHuman 2021 pathways database revealed that primary cell cultures expressed similar or equal levels of genes involved in EGFR tyrosine kinase inhibitor (TKI) resistance, PD-1-associated therapeutic pathways as well as regulatory pathways of purine metabolism, cellular proteostasis and hedgehog signaling (**Figure 5B**). Several of these genes encoded growth factors, including *PDGFA, PDGFC* and *PDGFD* as well as *VEGFA*. Among the genes associated with PD-1-based immunotherapy were *CD273* (coding for PD-L1), *IFNG, HLA-DRB1* and *PTPN11* (full list see **Supplementary Table 4**). In conclusion, primary cells expressed similar or equal levels of pathways crucial for current therapeutic approaches in the management of advanced ccRCC or metastasis.

### Expression of Hypoxia-Associated Genes and Neo-Angiogenesis in Primary Cell Cultures

Inactivating *VHL* mutations are characteristic for ccRCC and induce an upregulation and stabilization of hypoxia-associated gene products and a shift towards aerobic glycolysis, also known as the Warburg effect (29). To quantify the gene expression of few selected genes, qRT-PCR was performed for *VHL, HIF1A* and *CA9.* Since EGFR/TKI- resistance pathways as well as genes involved in PD-1 blockade were equally expressed in cell cultures compared to the tissue of origin, we performed additional analyses for the mRNA expression of *VEGFA, VEGFC*, *EGFR* and *PD-L1*. Immunohistochemistry was performed to verify the protein expression *in vivo* as well as *in vitro*.

**Figure 6:**
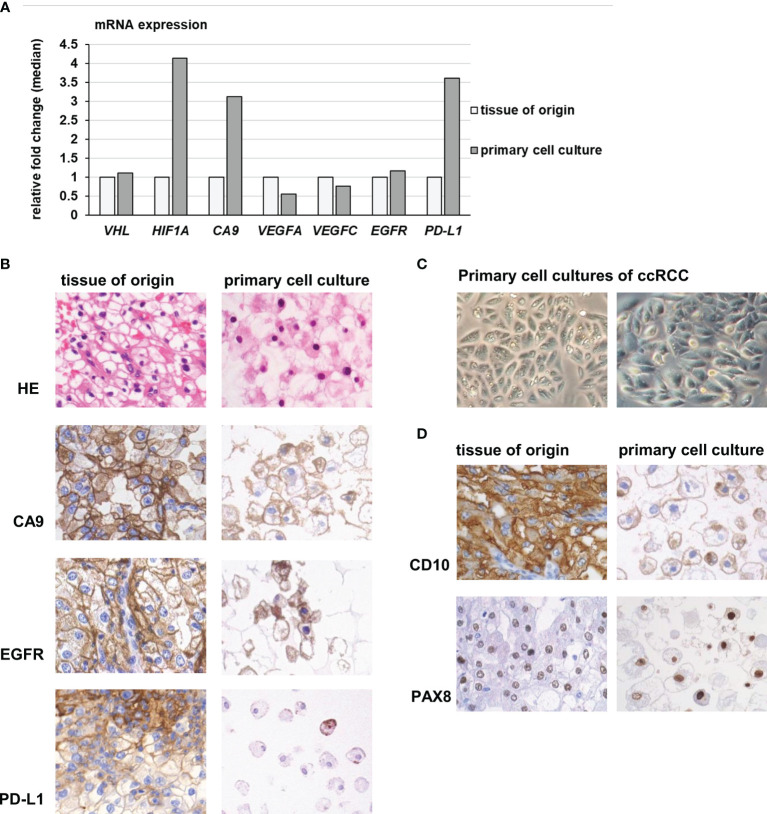
Gene expression of hypoxia- and angiogenesis related genes in primary cell cultures. **(A)** Primary cell cultures of ccRCC had slightly increased mRNA expression levels of HIF-1A and CA9, compared to the tissue of origin. The mRNA expression of VHL, VEGFA, VEGFC and EGFR remained stable, while *in vitro* a slightly increased expression of PD-L1 was observed. **(B)** Primary cell cultures expressed CA9, EGFR and PD-L1 in accordance to the tissue they derived from. Especially PD-L1 protein was only partially expressed in small, isolated cell nests in tumor tissue and only in some of the primary cells (magnification 400 x). **(C)** The cell cultures grew in characteristic epithelial nests and sheets of large polygonal tumor cells (magnification 400 x). **(D)** The primary cell cultures as well as the tumor tissue they derived from expressed CD10, another characteristic marker of ccRCC, as well as PAX8, proving their renal-epithelial origin (magnification 400x).

The ccRCC primary cell cultures showed similar mRNA expression levels of *VHL* and slightly higher levels of *HIF1A* and *CA9* than the tissue of origin (**Figure 6A**). A strong protein expression of *CA9* was detected in ccRCC primary cell cultures as well as in the tissue of origin (**Figure 6B**). As Enrichr analysis of the equally expressed pathways indicated (see above), the mRNA expression of the neo-angiogenetic proteins *VEGFA* and *VEGFC* as well as *EGFR* remained stable under *in vitro* conditions. As reported before (16), the tumor cells shared the same *VHL* mutations as the tumor tissue they derived from (**Supplementary Table 5**). Five of the eight ccRCC tumors included in this study harbored *VHL* mutations. Two of these five mutations (ccRCC 3: heterozygous frameshift deletion; ccRCC4: heterozygous in frame deletion) were not described in COSMIC database, but in our previous publication (see above). The *HIF1A* expression compared to the benign renal tissue was partially elevated, but not linked to specific *VHL* mutations (data not shown). Furthermore, the *VHL* mutation status was not linked to increased expression levels of *HIF1A* compared to the tissue of origin.

When compared directly with the corresponding tissue of origin, a slight upregulation of *PD-L1* was noticed *in vitro* compared to the tumor tissue (fold change median 3.6, **Figure 6A**). However, primary cells as well as tumor tissue of origin only partially expressed PD-L1 protein in small, isolated cell nests. A difference between tumor tissue and derived cell cultures was not observed.

To verify the tumor origin of the primary cell cultures, we regularly inspected the cells *in vitro:* They displayed the typical growth behavior of epithelial nest formation, consisting of polygonal, large tumor cells with prominent nucleoli and plentiful cytoplasm (**Figure 6C**).

Besides CA9, the tumor cells additionally expressed CD10, another marker of ccRCC, and PAX8, proving their renal epithelial origin. The protein expression matched the tumor tissue of origin (**Figure 6D**).

## Discussion

Many commercial tumor cell lines of renal cancer used all over the world have been proven to be problematic: For many of them, the histologic tumor entity has been obscured due to a lack of classification at the time of generation and accumulated genetic changes over many passages (30–32).

Primary cell cultures, 2-dimensional as well as 3-dimensional organoid approaches, allegedly remain closer to the tissue of origin and therefore are an important, more representative tool for *in vitro* experiments. Current studies revealed a closer genetic relationship with the tissue they derived from, with only a few genetic alterations on the genomic level, and an expression of characteristic marker proteins matching the tumor tissue (10, 12, 13, 16). However, the information about mRNA expression and adaptation to the cell culture conditions is still very limited.

We therefore established and characterized primary cell lines from ccRCC and matched non-neoplastic tissue as reported before (16). The following 3’mRNA sequencing analysis of primary cell cultures compared to the tumor tissue of origin revealed over 20.000 genes being differentially expressed. Among the top 50 dysregulated genes were important regulators of cell metabolism such as *PDK4, ALDOB* and others. Aldolases A, B and C all are involved in glycolysis and gluconeogenesis and are often dysregulated in tumor cells: In renal cancer, aldolase B is usually downregulated, in contrast to benign renal tissue, in which it is additionally expressed (33). However, a recent study revealed that the overexpression of aldolase A in RCC is associated with metastasis and a downregulation, although performed on commercial cell lines, suppressed invasion, and proliferation (34). Aldolase B was shown to be also overexpressed in other tumors like colorectal adenocarcinoma, promoting progression (35). Other genes among the top 50 dysregulated genes were involved in cell adhesion, angiogenesis, immune signaling and interaction with the extracellular matrix.

Since a detailed analysis of each of these genes would go beyond the scope of our objectives and to narrow down the genetic alterations on pathways, we performed a gene set enrichment analysis (GSEA) as well as Enrichr analyses for the enriched as well as up- and downregulated pathways.

According to the GSEA, primary cell cultures displayed an upregulation of cell cycle, DNA repair, MTORC1 signaling, but also metabolic pathways like glycolysis. A reversion from oxidative metabolism and gluconeogenesis to high rates of glycolysis has already been described for non-neoplastic renal cell cultures and might be explained by limited oxygen diffusion in cell culture medium (36, 37). The Enrichr pathway assignment analysis (KEGG 2021 Human database) additionally revealed enrichment of chemokine encoding genes (*CXCL1, CXCL2, CXCL3, CXCL6, CXCL5, CXCL8, CCL2*) which were aligned to several pathways like amoebiasis and rheumatoid arthritis. This pathway alignment, however, obscured the broader function of these genes, which are key regulators of angiogenesis, tumor growth and proliferation and tumor inflammation (38).

Among the upregulated pathways was also the *IL-17* pathway, which, besides inflammation and immune regulation, also involves the MAP kinase signaling and the chemokines mentioned above (39). To summarize our findings, we observed an upregulation of key pathways involved in angiogenesis, cell proliferation signaling, chemokine signaling as well as metabolic changes *in vitro.*

The Enrichr analyses revealed a downregulation of genes regulating cell adhesion and immune differentiation of T helper cells, which could be explained by the absence of the immune cell compartment of the tissue of origin as well as the process of cell separation during cultivation. Additionally, pathways of the hematopoietic cell lineage were downregulated, which can also be explained by the absence of blood cells.

When using primary cell cultures as an *in vitro* model for the development of targeted therapies, it is crucial to recognize their potential as well as their limitations. We therefore additionally analyzed the pathways, which were similarly or equally expressed in tumor tissue and cell cultures: Interestingly, the analysis revealed an equal expression of genes crucial in the development and progression of renal cancer, such as *HIF1A* and *VEGFA*. RCC, especially the clear cell subtype, highly relies on aerobic glycolysis despite the presence of oxygen, and therefore promotes a pseudo-hypoxic gene expression: This circumstance is known as the Warburg-effect and has been investigated over decades (40). The aerobic glycolysis could be a potential therapeutic target and has been investigated before by our group as well as other researchers (16, 41, 42). The pseudo-hypoxic metabolism of clear cell renal cancer is based on a loss of *VHL* function, which induces stabilization and upregulation of hypoxia-associated gene products like *HIF1A* and *VEGF* (29). Interestingly, when compared to their individual tumor of origin and not as a cohort, primary cell cultures displayed an even slightly higher mRNA expression of *CA9* and *HIF1A*, while the expression of *VHL, VEGFA* and *VEGFC* remained constant. This indicates an even stronger shift towards aerobic glycolysis and pseudo-hypoxia under cell culture conditions. Additionally, real hypoxia might as well explain this increase partially: Surprisingly, studies observed signs of hypoxia in renal epithelial cell cultures even when the pO_2_ levels, temperature (37°C) and saturation of 95% O_2/_5% CO_2_ corresponded to the physiologic blood conditions *in vivo* (36, 43). Additionally, a loss of cell differentiation in favor of proliferation was described. This dedifferentiation includes loss of mitochondria, an increase of enzyme expression of anaerobic glycolysis as well as loss of microvilli and ruffled membrane structure, a process observed for tumor cells and renal epithelial cells (7, 43, 44). We can therefore assume that the increased expression of hypoxia-associated genes is a reaction to the change of the microenvironment.

These changes were also observed in another study, generating primary cell cultures of benign hepatocytes: Cassim et al. described severe metabolic changes in mitochondrial respiratory capacity during cell isolation and cultivation as well as a reduction of antioxidative-related metabolites (45). The latter observation reveals another interesting aspect: Not only basic cellular pathways like metabolism and respiratory chain regulation may be alternated *in vitro*, but also organ-specific, cancer-specific or even tumor-subtype-specific cellular characteristics. The importance of taking these changes into consideration when performing experimental research cannot be overemphasized.

Two other pathways were similarly expressed in primary cell cultures and in tumor tissue of origin, and these hold crucial implications: The *EGFR* tyrosine kinase resistance pathway as well as well as the *PD-1* pathway.

Because of its resistance to conventional chemotherapy and radiation, RCC remains an insidious and challenging disease. 20% to 30% of patients present with metastatic disease at first diagnosis, additional 30% develop metastases later (46). Although progress has been made in recent years with the constant and ongoing search for optimized immunotherapies, many patients do not respond to these new therapeutics at all or still succumb to their disease later (47, 48). Our WikiPathway 2021 Human analysis displayed that the gene pathways targeted by tyrosine kinase inhibitors such as axitinib, cabozantinib and pazopanib as well as immune checkpoint inhibitors do not change under *in vitro* conditions. This implicates, that primary cell cultures are a feasible model for further investigations of the pathological mechanisms of resistance development or absent response to the still limited arsenal of therapeutics.

As reported before, we already demonstrated that primary cell cultures can be used to investigate therapeutic approaches *in vitro*, using a glycolysis inhibitor in combination with commonly used tyrosine kinase inhibitors on several RCC entities (16). We also tested immune checkpoint inhibitors and the targeted therapy agent cabozantinib on three-dimensional organoids *in vitro* (49). In this study, we once more emphasize not only the close relationship to the tumor tissue regarding cell metabolism and (pseudo)hypoxia, but also demonstrated the molecular similarity in relation to the expression of pathways holding therapeutic relevance.

However, since several studies reported that also HIF1A and HIF2A can induce upregulation of *PD-L1* (50, 51), the interaction between (pseudo)hypoxic signaling and expression of certain immune pathways must be further elucidated.

## Conclusion

In this study, we extensively characterized and explored mRNA expression changes in primary cell cultures compared to the tissue they derived from and assigned the differences as well as similarly expressed genes to central pathways in tumor cell biology. We furthermore validated the results of several genes involved in adaptation to hypoxia, growth factor expression and immune evasion pathways. Our data demonstrated and confirmed already described changes in the metabolic pathways under cell culture conditions, but also explored several up- or downregulated pathways, which are commonly neglected when using primary cells in experimental research. Interestingly, primary cells express many genes involved in immune evasion and growth factor signaling very similarly or equally to the tumor tissue they derived from, making them a feasible and very important tool for specified translational and experimental research. Our results also illustrate the complex and numerous interactions between the adaptive pathways and allow new translational approaches in the treatment of renal cancer. The differences, as well as the common features between the primary cell cultures and the tissue they derived from, must be considered in every research project using them as *in vitro* model.

## Data Availability Statement

The datasets presented in this study can be found in online repositories. The names of the repository/repositories and accession number(s) can be found below: https://www.ncbi.nlm.nih.gov/sra/PRJNA803031.

## Ethics Statement

The studies involving human participants were reviewed and approved by Ethics Committee at Bonn University Hospital. The patients/participants provided their written informed consent to participate in this study.

## Author Contributions

Conceptualization, AGS, LKE, and MIT; methodology, LKE, AGS, TM, and MM; validation, LKE, AGS, JE, and MR; formal analysis, AGS, LKE, TM, MM, GK, and MIT; investigation, AGS and LKE; data curation, AGS, LKE, MIT, and TM; writing—original draft preparation, AGS, LKE, and MIT; writing—review and editing, AGS, LKE, JE, GK, MR, TM, MM, and MIT; visualization, AGS and LKE; supervision, MIT; project administration, MIT; funding acquisition, MIT and GK. All authors have read and agreed to the published version of the manuscript.

## Conflict of Interest

The authors declare that the research was conducted in the absence of any commercial or financial relationships that could be construed as a potential conflict of interest.

## Publisher’s Note

All claims expressed in this article are solely those of the authors and do not necessarily represent those of their affiliated organizations, or those of the publisher, the editors and the reviewers. Any product that may be evaluated in this article, or claim that may be made by its manufacturer, is not guaranteed or endorsed by the publisher.
