# Supplementary figures and images for: RNA Sequencing Reveals Alterations and Similarities in Cell Metabolism, Hypoxia and Immune Evasion in Primary Cell Cultures of Clear Cell Renal Cell Carcinoma
Source: Front Oncol. 2022 May 11;12:883195. doi: 10.3389/fonc.2022.883195 (PMC9130782; doi:10.3389/fonc.2022.883195)

## Supplementary Figure: Workflow

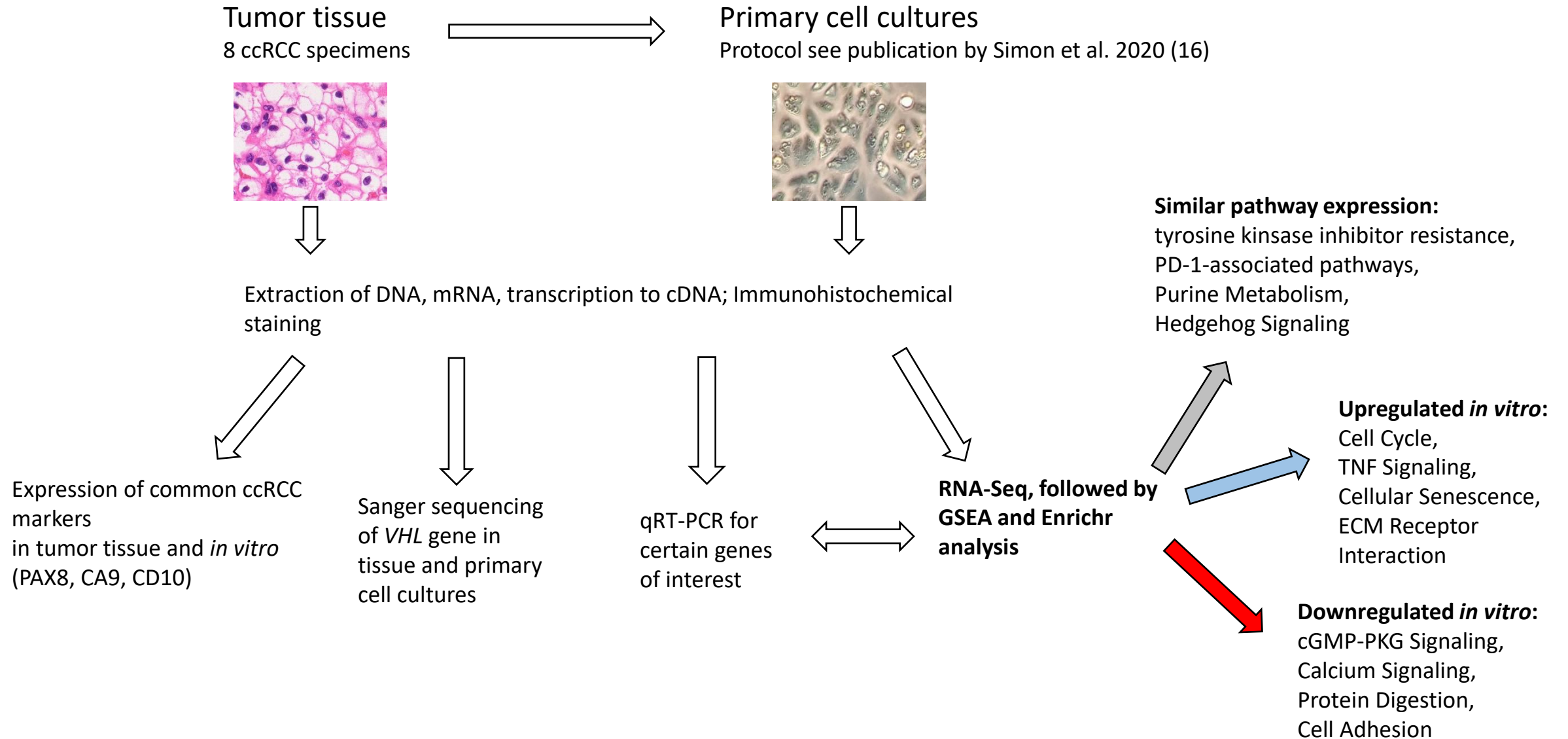

Supplement: Supplementary file 1 [file Image_1.pdf]
