# Supplementary material for: RNA Sequencing Reveals Alterations and Similarities in Cell Metabolism, Hypoxia and Immune Evasion in Primary Cell Cultures of Clear Cell Renal Cell Carcinoma
Source: Front Oncol. 2022 May 11;12:883195. doi: 10.3389/fonc.2022.883195 (PMC9130782; doi:10.3389/fonc.2022.883195)
Supplement: Supplementary file 2 [file Table_1.docx]

| **Supplementary table 1:** Primers of quantitative real time PCR for gene expression in primary cell cultures of ccRCC | | | |
| --- | --- | --- | --- |
| **primer** | **sequence 5‘ - 3‘** | **efficiency absolute** | **efficiency [%]** |
| CA9_qPCR_Fw. | GCTAGAGATGGCTCTGGGTC | 1.926 | 92.6 |
| CA9_qPCR_Rv. | CTGAGCCTTCCTCAGCGATT |  |  |
| EGFR-1_qPCR_Fw. | AAATCATCTGTGCCCAGCAGT | 2.01 | 101.0 |
| EGFR-1_qPCR_Rv. | CTTCGTCTCGGAATTTGCGG |  |  |
| HIF-1a_qPCR_Fw. | GCCGCTGGAGACACAATCAT | 2.017 | 101.7 |
| HIF-1a_qPCR_Rv. | CGTTTCAGCGGTGGGTAATG |  |  |
| PARK2_qPCR_Fw. | TAGCTTTGCACCTGATCGCA | 1.959 | 95.9 |
| PARK2_qPCR_Rv. | CCACACAAGGCAGGGAGTAG |  |  |
| PDL-1_qpCR_Fw. | CCTGCAGGGCATTCCAGAAA | 2.00 | 100.0 |
| PDL-1_qPCR_Rv. | TAGGTCCTTGGGAACCGTGA |  |  |
| PINK1_qPCR_Fw. | CATGCCTACATTGCCCCAGA | 1.972 | 97.2 |
| PINK1_qPCR_Rv. | TGACTGCTCCATACTCCCCA |  |  |
| PPIA__qPCR_Fw. | TGGTATAAAAGGGGCGGGAG | 1.998 | 99.8 |
| PPIA_qPCR_Rv. | AAGAACACGGTGGGGTTGA |  |  |
| VEGFR-A_qPCR_Fw. | ATCAGTTCGAGGAAAGGGAAA | 1.974 | 97.4 |
| VEGFR-A_qPCR_Rv. | CATTTACACGTCTGCGGATCT |  |  |
| VEGFR-C_qPCR_Fw. | TTGCCAATCACACTTCCTGC | 1.976 | 97.6 |
| VEGFR-C_qPCR_Rv.. | CCATCTGTTGAGTCATCTCC |  |  |
| VHL_qPCR_Fw. | CCGTATGGCTCAACTTCGAC | 1.903 | 90.3 |
| VHL_qPCR_Rv. | CTGACGATGTCCAGTCTCCTG |  |  |
| All primers were tested for efficiency using a dilution row (1, 1:2, 1:4, 1:8, 1:16 etc.). A complete duplication of cDNA samples generated an efficiency of 2.0 (100 %). In case of HIF-1A and EGFR-1, very few primers formed primer-primer complexes, falsely giving a slightly higher efficiency than 2.0.  The qPCR reagent sample consisted of: iTaq Universal SYBR Green Super Mix (2x) Bio-Rad (5 µL), primer forward (10 µM) (0.4 µL), primer reverse (10 µM) (0.4 µL), H_2_O dest. (3.2 µL), 1.0 µL transcribed cDNA.  The qPCR was performed using a ViiA 7 Real-Time PCR System (Applied Biosystems).  The conditions were: 25 °C to 50 °C heat up, 50 °C (2 min); activation of iTaq polymerasis at 95 °C (10 min); 50 cycles: 95 °C (15 s), 63 °C (2 s), 60 °C (60 s); melting curve analysis: heat up to 95 °C with 0.05 °C/s, 95 °C (30 s), 60 °C (infinitive). | | | |
