# Supplementary material for: RNA Sequencing Reveals Alterations and Similarities in Cell Metabolism, Hypoxia and Immune Evasion in Primary Cell Cultures of Clear Cell Renal Cell Carcinoma
Source: Front Oncol. 2022 May 11;12:883195. doi: 10.3389/fonc.2022.883195 (PMC9130782; doi:10.3389/fonc.2022.883195)
Supplement: Supplementary file 3 [file Table_2.docx]

| antibody | clone | manufacturer | species | dilution |
| --- | --- | --- | --- | --- |
| CA9 | ab15086 | Abcam | rabbit | 1:8000 |
| EGFR | E30 | Agilent | mouse | 1:50 |
| PD-L1 | ZR-3 | Zeta | rabbit | 1:50 |
| PAX8 | MRQ-50 | CellMark | mouse | 1:100 |
| CD10 | SP67 | Ventana | rabbit | ready to use |
