# Supplementary material for: RNA Sequencing Reveals Alterations and Similarities in Cell Metabolism, Hypoxia and Immune Evasion in Primary Cell Cultures of Clear Cell Renal Cell Carcinoma
Source: Front Oncol. 2022 May 11;12:883195. doi: 10.3389/fonc.2022.883195 (PMC9130782; doi:10.3389/fonc.2022.883195)
Supplement: Supplementary file 6 [file Table_5.docx]

| **supplementary table 5.** *VHL* mutations in ccRCC specimens | | | | |
| --- | --- | --- | --- | --- |
| tumor | mutation  (DNA sequence) | mutation  (amino acid sequence) | type | COSMIC-ID |
| ccRCC1 | c.491 A>T | p.Q164L | Substitution (Missense) | COSM30288 |
| ccRCC3 | c.420_439del20/+ | p.N141fs25*/+ | Deletion (Frameshift) | - * |
| ccRCC4 | c.223_225delCAT | p.I75del/+ | Deletion  (in frame) | - * |
| ccRCC7 | c.287A>C | p.Q96P | Substitution (Missense) | COSM144717 |
| ccRCC8 | c.269A>T | p.N90I | Substitution (Missense) | COSM14390 |
| The mutations were described before, partially in COMSIC database, partially in our previous publication (*) (Simon et al. 2020 (16), different tumor ID’s were annotated in this publication due to newly included tumor specimens and order of experimental inclusion). | | | | |
